# Basal Proliferation and Acantholysis May Represent Histological High-Risk Factors for Progression into Invasive Squamous Cell Carcinoma: A Comparison Study in Solid Organ Transplant Recipients and Matched Immunocompetent Patients

**DOI:** 10.3390/cancers15061765

**Published:** 2023-03-14

**Authors:** Conrad Falkenberg, Thomas Dirschka, Georgia Gilbert, Eggert Stockfleth, Bernhard Homey, Lutz Schmitz

**Affiliations:** 1 Department of Dermatology, University Hospital Düsseldorf, Medical Faculty, Heinrich-Heine-University, 40225 Düsseldorf, Germany; 2 Faculty of Health, University Witten-Herdecke, Alfred-Herrhausen-Straße 50, 58448 Witten, Germany; 3 CentroDerm Clinic, Heinz-Fangman-Straße 57, 42287 Wuppertal, Germany; 4 Edinburgh Medical School, The University of Edinburgh, Edinburgh EH16 4SB, UK; 5 Department of Dermatology, Venereology and Allergology, Ruhr-University, 44780 Bochum, Germany

**Keywords:** acantholysis, actinic keratosis, basal proliferation, immunosuppression, cutaneous squamous cell carcinoma, solid organ transplant recipients

## Abstract

**Simple Summary:**

AKs restricted to the lower third of the epidermis (AK I), with marked basal growth patterns (PRO III) and acantholysis, are associated with an increased risk of progression to invasive squamous cell carcinoma (iSCC). To confirm that these are high-risk histological features for tumour progression, we compared AKs from solid organ transplant recipients (sOTRs), known to carry an increased risk for progression to iSCC, to a matched immunocompetent control group (ICG). We assessed histological grading (AK I-III), basal growth patterns (PRO I-III) and the presence of acantholysis. The AKs from sOTRs showed significantly more AKs graded as AK I and PRO III compared to the ICG. Acantholysis was significantly more frequent in sOTRs and acantholytic AKs were significantly associated with advanced basal proliferation. Thus, AKs with marked basal proliferation and acantholysis may represent histological high-risk factors for progression into iSCC.

**Abstract:**

Histological risk factors of AKs cannot be directly determined. Recent studies indicate that AKs restricted to the lower third of the epidermis (AK I), with marked basal proliferation (PRO III) and acantholysis, are associated with an increased risk of progression to invasive squamous cell carcinoma (iSCC). To confirm the aforementioned histological risk factors, this study compared AKs from solid organ transplant recipients (sOTRs), known to carry an up to 250-fold higher risk for progression into iSCC, to a matched immunocompetent control group (ICG). In total, 111 AKs from 43 sOTRs showed more AKs (*n* = 54, 48.7%) graded as AK I compared to 35 AKs (31.5%) in the ICG (*p* = 0.009). In line with these findings, 89 AKs (80.2%) from sOTRs showed pronounced basal proliferation (PRO III) compared to 37 AKs (33.3%) in the ICG (*p* < 0.0001). Acantholysis was more frequent in sOTRs than the ICG (59.5% vs. 32.4%, *p* < 0.0001) and more frequently associated with advanced basal proliferation (*p* < 0.0001). In conclusion, this study showed that acantholytic AKs graded as AK I and PRO III are predominantly found in a population at high risk of iSCC. Thus, AKs with marked basal proliferation and acantholysis should be assumed to be histological high-risk factors for the progression into iSCC.

## 1. Introduction

Actinic keratoses (AKs) are regarded as early in situ squamous cell carcinoma that can progress into invasive cutaneous squamous cell carcinoma (iSCC), and subsequently metastasise in approximately 4% of cases [1,2,3,4]. AKs are predominantly found on UV-exposed skin and the overall prevalence is estimated to range between 6% and 26% [5,6]. Due to an ageing population and changed leisure activities in industrialised areas, incidences are expected to rise in the next few decades [5,6]. Immunosuppression constitutes an additional risk factor for developing AKs. Due to immunosuppressant medications, solid organ transplant recipients (sOTRs) carry a 250-fold increased risk of developing AKs and a 65 to 250-fold increased risk of developing iSCC. The prevalence rises with increasing duration and intensity of immunosuppression [7,8,9,10,11,12]. Around 10% (0.025–16%) of all patients with AKs develop iSCC, in contrast to around 30% of patients with additional immunosuppression [3,7].

The difference between in situ and invasive growth patterns can be histologically assessed. The established histological classification scheme based on the growth pattern of atypical keratinocytes throughout the epidermis by Roewert-Huber et al. (AK I-III) does not predict the risk of progression to iSCC. Particularly AKs restricted to the lower third of the epidermis (AK I) are most commonly found adjacent to iSCC [13,14], which indicates the lowest grading in terms of this classification. In contrast, a recently proposed classification (PRO I-III) evaluates the downward-directed growth pattern of AKs and may be promising in terms of risk stratification [15,16]. Recent studies indicate that a marked basal growth pattern might be associated with an increased risk of progression [13,15,16]. Independent of the downward-directed growth pattern, acantholysis seems to be associated with an increased risk of progression to iSCC. In a study that compared AKs that were refractory to treatment and treatment-naïve AKs, the treatment-resistant AKs showed significantly more acantholysis and marked basal proliferation (PRO III). Acantholysis was also correlated with increasing basal proliferation of atypical keratinocytes, irrespective of treatment resistance [17].

It is important to identify AKs with a high potential of progression into invasive tumour for several reasons. Firstly, AKs and the surrounding actinically damaged fields are typically treated to prevent the evolution of iSCC. This may lead to overtreatment and high associated costs and a significant burden for the patients [18,19,20]. Secondly, early identification of the predictive markers for AKs would allow practitioners to focus on patients with an increased risk of iSCC and in turn prevent life-threatening disease.

Histological risk factors for the progression from AK to iSCC cannot be directly determined, as it is impossible to know when or if an AK would have become invasive once the lesion is excised. Recent studies suggest that AKs restricted to the lower third of the epidermis (AK I), with marked basal proliferation (PRO III) and acantholysis, are associated with an increased risk of progression to iSCC [13,14,15,16,17]. The evaluation of AKs from a population with a known high risk for progression into iSCC could substantiate the aforementioned histological risk factors. SOTRs carry an increased risk of developing AKs and these AKs progress more rapidly into iSCC [7,8,9,10,11,12,21]. They may, therefore, represent a cohort with histological risk factors associated with the progression from AK to iSCC. Thus, this study aims to compare the histological characteristics of AK samples from sOTRs to a matched immunocompetent control group (ICG). Histological differences between these groups are likely to indicate high risk features of the progression from AK to iSCC.

## 2. Materials and Methods

### 2.1. Study Population

Cases were retrospectively selected from the Skin Cancer Center of Heinrich-Heine-University Düsseldorf (Düsseldorf, Germany) database, in the period January 2008–June 2021. The study was approved by the institution’s ethics committee (no. 2021-1620) and was conducted according to the Declaration of Helsinki. The biopsies were performed for various reasons (e.g., to confirm the clinical diagnosis, to rule out iSCC or as single-lesion treatment). In total, 111 AKs from sOTRs were identified and eligible for histological analysis. Subsequently, we identified a matched cohort of histologically diagnosed AKs in patients who were not immunosuppressed from the same period of time. To ensure regional homogeneity for comparison, AKs were selected from patients with the same age, gender and anatomical region (e.g., head and trunk). Samples of inadequate quality (e.g., torn tissue and no dermal tissue captured) were excluded from this study.

### 2.2. Microscopic Evaluation

All haematoxylin and eosin (H&E)-stained sections (3 µm of thickness) were analysed at scanning magnification and at 20- fold magnification. Two independent investigators (LS and CF) classified the samples as per their upward projected growth pattern (AK I, II, or III), according to Roewert-Huber et al. [14]. The downward projected growth pattern was classified as PRO I, II or III according to Schmitz et al. [15]. In cases of different grades within the same lesion, the highest grade was chosen. Disagreements between the two investigators were discussed and resolved using a double-headed microscope.

The basal growth pattern was evaluated as showing no criteria of increased basal growth (PRO 0), crowding of basal atypical keratinocytes (PRO I), budding of atypical keratinocytes into the upper papillary dermis and formation of round nests of atypical keratinocytes (PRO II), or spiky or filiform papillary elongation of atypical keratinocytes protruding into the upper dermis and exceeding the thickness of the overlying epidermis (PRO III, papillary sprouting) [15]. The upward projected growth pattern was evaluated as atypical keratinocytes limited to the lower third of the epidermis (AK I), atypical keratinocytes extending to the lower two thirds of the epidermis (AK II) or full-thickness atypia of the epidermis (AK III) [14].

The underlying inflammation, degree of hyperkeratosis and amount of solar elastosis were semi-quantitatively classified into none, mild, moderate, severe and very severe [22]. Acantholysis and the follicular involvement of hair follicles (including the associated sebaceous gland) were investigated and dichotomously evaluated, if present or not.

### 2.3. Statistical Analysis

Data were analysed using the SPSS software version 26.0 (IBM, Armonk, NY, USA). The distribution of the data was assessed using the Shapiro–Wilk test. In case of normal distribution, data were expressed as mean and standard deviation (SD); if not, data were expressed as median and interquartile range (IQR). Data were statistically analysed using the Mann–Whitney U test for unpaired samples. *p*-values < 0.05 were considered to be statistically significant.

## 3. Results

In total, 111 AKs derived from 43 sOTRs were included in this study with a median number of 2 AKs per patient. The majority of patients were male (*n* = 34, 79.1%) and the mean age was 69.3 (±8.1) years. There were no differences in age and gender between the sOTRs and ICG due to matching. In total, 35 patients (81.4%) received a kidney transplant, 7 patients (16.3%) received a heart transplant and 1 (2.3%) patient underwent both kidney and heart transplantation. In addition, 37 patients had undergone one transplant (86%), 4 patients received two transplants (9.3%) and 2 patients underwent three transplants (4.7%). The median number of years taking immunosuppressant medication was 11 (7–20.5). Overall, 39 patients (88.6%) had a positive history of non-melanoma skin cancer. Further patient characteristics are shown in Table 1.

AKs from sOTRs showed lower AK I-III grading, with 54 AKs (48.7%) graded as AK I, 38 (34.2%) as AK II and 19 (17.1%) as AK III. In comparison, in the ICG, 35 AKs (31.5%) were graded as AK I, 46 (41.4%) as AK II and 30 (27.0%) as AK III, respectively (*p* = 0.008). Conversely, more AKs derived from sOTRs showed pronounced basal proliferation. In this group, 1 AK (0.9%) was classified as PRO 0, 5 (4.5%) as PRO I, 16 (14.4%) as PRO II and 89 (80.2%) as PRO III, compared to 9 AKs (8.1%) graded as PRO 0, 27 (24.3%) as PRO I, 38 (34.2%) as PRO II and 37 (33.3%) as PRO III in the ICG, respectively (*p* < 0.0001). Acantholysis was more frequent in sOTRs than in the ICG (59.5% vs. 32.4%, *p* < 0.0001). In addition, a higher number of AKs from sOTRs had follicular involvement (83.8% vs. 69.4%, *p* < 0.0001). AKs in the sOTR group showed a higher degree of hyperkeratosis (2.27 ± 0.99 vs. 1.7 ± 1.08, *p* < 0.0001). Conversely, the degree of solar elastosis was higher in AKs from the ICG (2.82 ± 1.02 vs. 2.36 ± 1.31, *p* = 0.014). There was no difference in the intensity of the infiltrate between sOTRs and the ICG (1.82 ± 0.91 vs. 1.83 ± 0.81, *p* = 0.437). Table 2 provides an overview of the differences between groups.

To investigate whether acantholysis was associated with the basal proliferation of AKs, all acantholytic AKs were pooled, irrespective of the immunosuppression status of the patient (*n* = 102) and compared to AKs without acantholysis (*n* = 120). Basal proliferation was more pronounced in acantholytic AKs. In total, 2 acantholytic AKs (1.9%) were graded as PRO 0, 8 AKs (7.8%) as PRO I, 20 AKs (19.6%) as PRO II and 72 AKs (70.6%) as PRO III. In contrast, in AKs without acantholysis, 8 AKs (6.7%) were classified as PRO 0, 25 AKs (20.8%) as PRO I, 34 AKs (28.3%) as PRO II and 53 AKs (44.2%) as PRO III, respectively (*p* < 0.0001).

## 4. Discussion

**Figure 1:**
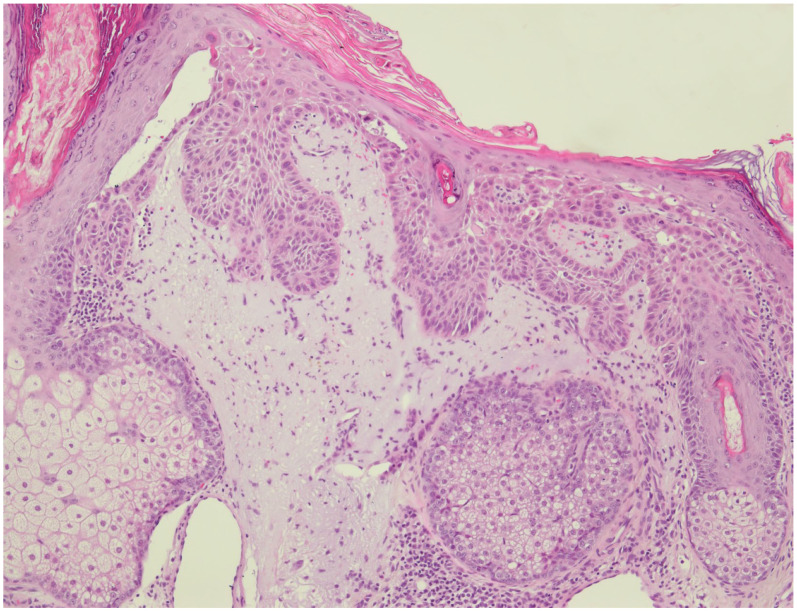
Histological section (H&E; original magnification ×40) of an actinic keratosis (AK) derived from a solid organ transplant recipient (sOTR) showing marked basal proliferation (PRO III), acantholysis, follicular involvement and pronounced solar elastosis. Marked basal proliferation (PRO III) and acantholysis were more prevalent in AKs from sOTRs than in the immunocompetent control group (*p* < 0.0001 and *p* < 0.0001, respectively).

This study shows that certain histological criteria can be used to risk stratify AKs. We found significantly more advanced basal proliferation (PRO III) and acantholysis in AKs from immunosuppressed sOTRs than in the ICG (Figure 1). Since sOTRs carry an up to 250-fold higher risk of developing iSCC, these results corroborate the recent findings that AKs with advanced basal proliferation are more common in the epidermis adjacent to iSCC [7,8,9,10,11,12,16]. Moreover, the majority of AKs derived from sOTRs had atypical keratinocytes restricted to the lower third of the epidermis (AK I). In contrast, full-thickness atypia of keratinocytes (AK III) was more prevalent in the ICG. Fernandez-Figueras et al. have previously shown that AKs with atypical keratinocytes restricted to the lower third of the epidermis are most commonly found in the adjacent epidermis of iSCC [13]. Thus, basal proliferation may be of greater predictive value in the emergence of iSCC than the extent of atypical keratinocytes spreading through the epidermal compartment. Therefore, basal proliferation grading could be used to histologically stratify the progression risk of single lesions.

Once an AK is excised, it is impossible to predict how the lesion would have developed (e.g., when and if it would have progressed into iSCC). Even excising a small portion from a large AK would likely alter the pathophysiology of the lesion, due to the inflammation caused by the invasive procedure and further analysis of its development would be biased. Since AKs in sOTRs progress more rapidly into iSCC, it may be inferred that AKs from sOTRs exhibit histological high-risk features [7,8,9,10,11,12,21]. Thus, if longitudinal data on the development of single lesions cannot be studied directly, investigating the histological characteristics of AKs in a high-risk population, such as sOTRs, may corroborate the recently published risk factors.

Acantholysis was significantly more frequent in AKs from sOTRs. A recent publication described that treatment-resistant AKs demonstrate more pronounced basal proliferation (PRO III), as well as acantholysis, than lesions that are treatment-naive. Acantholytic AKs also showed a significant correlation with increasing basal proliferation [17]. Thus, acantholysis may not only contribute to treatment resistance, but treatment-resistant lesions may also carry a higher risk for progression to iSCC. Our findings support the assumed role of acantholysis in the progression to iSCC. In our pooled analysis of all acantholytic AKs, we detected significantly more distinct basal proliferation in acantholytic AKs than in AKs without acantholysis. Therefore, acantholysis seems to be a high-risk histological feature for the progression of AK to iSCC. The loss of cell–cell adhesion constitutes one of the main features of the epithelial-to-mesenchymal transition of keratinocytes, which gain the potential to become invasive and subsequently metastasise [23,24,25]. Hence, acantholysis might contribute to the incipient invasive potential of AKs.

AKs from sOTRs showed a higher degree of hyperkeratosis compared to the ICG. This may be multifactorial, as not only do sOTRs carry an approximately 250-fold higher risk of developing AKs and iSCC than the overall population, these AKs also progress more rapidly into iSCC [7,8,9,10,11,12,21]. Thus, it is likely that the clinician does not excise every lesion due to their multitude, but instead selects the most hyperkeratotic lesions to rule out iSCC. Conversely, solar elastosis was unsurprisingly more pronounced in the ICG, likely due to the advice regarding the avoidance of UV exposure, which is provided to all patients undergoing solid organ transplantation. This may imply that the role of UV radiation is not as crucial in the pathogenesis of AKs in sOTRs as in the immunocompetent population. It is more likely that immunosuppressant medication leads to the emergence of AKs due to the impaired function of immune regulatory cells.

Previous studies indicate that the peritumoural infiltrate is more cellular in immunocompetent patients than in immunosuppressed patients [26,27]. Interestingly, in our semi-quantitative analysis of the intensity of the dermal infiltrate in AKs from sOTRs and immunocompetent patients, we found no difference. However, increased peritumoural infiltrate intensity has only been found in iSCC and research on the immune infiltrate in AKs from immunocompetent versus immunosuppressed patients is lacking. Unfortunately, we could not further investigate the composition of the immune infiltrate in our study due to a lack of immunohistochemically stained tissue.

In the tumour microenvironment (TME), malignant and non-malignant cells (e.g., leukocytes, macrophages, andfibroblasts) interact in a complex and dynamic manner. It is well established that the TME plays an important role in solid tumour promotion and progression [28,29,30]. In AKs with advanced basal proliferation (PRO II–PRO III), it is thought that stromal cells fail to adequately control the atypical keratinocytes in the epithelium, allowing basal layer protrusion into dermal tissue. This leads to the enlargement of the contact surface between the altered epithelium and dermal tissue and may subsequently depict a higher risk of progression into iSCC [16,17]. This is most likely due to an alteration of the TME, which is required for the progression from AK to iSCC. Immunosuppressant medication exacerbates this, due to the impaired immune surveillance and reduced eradication of precancerous lesions [12,31]. In contrast, the stromal cells may be able to effectively control the atypical keratinocytes in the case of an upward-directed growth pattern only (AK II–AK III), preventing basal layer protrusion, and thus representing a lower risk of progression. This may explain the more advanced basal proliferation in AKs from sOTRs that we found in this study. In light of this concept, basal proliferation and acantholysis may be histological features that indicate reduced stromal cell control.

However, a difference between sOTRs and the ICG still remained, despite matching for the patient’s age, gender and anatomical location. Lesions derived from sOTRs were more hyperkeratotic and were, therefore, likely excised to rule out iSCC, while lesions in the ICG were presumably excised with curative intent as a single lesion treatment. This selection bias could contribute to the histological differences between sOTRs and ICG. However, it has already been shown that conclusions about the histological characteristics of an AK lesion cannot reliably be drawn from its clinical appearance. In a recent study, the authors used the Olsen classification to grade AKs before excision, according to their degree of hyperkeratosis and thickness. They compared this to histological grading using the classification by Röwert-Huber et al. and concluded that the degree of hyperkeratosis and thickness of an AK lesion could not reliably assess the underlying histological grading [32]. Thus, it seems unlikely that the selection bias plays a major role in explaining the differences between the study groups.

To date, literature concerning the histological characteristics of AKs in sOTRs is scarce. Boyd et al. evaluated 30 randomly selected AKs from 25 sOTRs and compared the histological features to those of 50 AKs from 45 immunocompetent patients [33]. They found the presence of bacteria, confluent parakeratosis, hyperkeratosis, verrucous changes and increased numbers of mitoses to be more common in lesions derived from sOTRs. However, the degree of hyperkeratosis was no longer significantly different after stratifying the data for patient age. In our study, AKs from patients under immunosuppression also depicted a higher degree of hyperkeratosis when compared with matched immunocompetent patients. Whilst Boyd et al. found no differences in acantholysis, adnexal involvement, degree of inflammation or basal proliferation, our study found more AKs with acantholysis, follicular involvement and advanced basal proliferation in immunosuppressed patients. Conversely, neither study found the degree of inflammation to be different. However, it should be noted that the differences in these findings may be explained by Boyd et al. only assessing basal proliferation dichotomously and their comparably small sample size [33].

The current national and international guidelines on the treatment of AKs recommend treating every lesion to prevent the evolution of iSCC. Since between 15% and 63% of AKs regress without therapy, this may easily lead to overtreatment and strained healthcare systems [18,19]. Our results endorse the recent findings, which suggest that acantholysis and basal proliferation are important histological characteristics to consider in the risk stratification of AKs and could, therefore, help rationalise treatment strategies for individual patients [16,17].

The main limitations of this study are the relatively small number of histological samples included, the retrospective study design, recruitment from only one centre and selection bias. In addition, the phototype of the patients and history of skin cancer in the ICG could not be obtained because it was not recorded in the clinical data. Due to the small number of sOTRs included and the heterogenous immunosuppressive regimens, valid comparisons between different types of solid organ transplants and different treatment regimens could not be performed. The underlying infiltrate was also only assessed semi-quantitatively without further characterisation of its composition, due to the lack of immunohistochemically stained tissue.

## 5. Conclusions

In conclusion, this study showed that AKs with atypical keratinocytes restricted to the lower third of the epidermis (AK I), pronounced basal proliferation (PRO III) and acantholysis are predominantly found in a population at high risk of progression from AK to iSCC. Marked basal proliferation and acantholysis may, therefore, represent histological high-risk factors for the progression from AK to iSCC.

## Figures and Tables

**Table 1:** Demographic and clinical characteristics of solid organ transplant recipients (*n* = 43).

Characteristic		*n*	(%)
Sex	Male	34	(79.1)
	Female	9	(20.9)
Age, years		69.3	(8.1) *
Solid organ transplant	Kidney	35	(81.4)
	Heart	7	(16.3)
	Kidney + heart	1	(2.3)
Number of transplants received per patient	1	37	(86.0)
	2	4	(9.3)
	3	2	(4.7)
Duration of immunosuppression, years		11	(7–20.5) *^#^*
	<5 years	9	(20.9)
	5–10 years	9	(20.9)
	>10 years	25	(58.1)
Immunosuppressant medication	Single/dual therapy	27	(62.8)
	Triple therapy	16	(37.2)
History of invasive skin cancer	Any type of skin cancer	39	(88.6)
	Non-melanoma skin cancer	39	(88.6)

* Data are expressed as mean (standard deviation). *^#^* Data are expressed as median (interquartile range).

**Table 2:** Histological characteristics of actinic keratoses (AKs) from solid organ transplant recipients (sOTRs) and matched immunocompetent control group (ICG).

Histological Characteristics	Overall (N = 222)	sOTRs (N = 111)	ICG (N = 111)	*p*-Value (sOTRs vs. Controls)
	n (%)	n (%)	n (%)	
AK Histological Severity				0.0078 *
AK I	89 (40)	54 (48.6)	35 (31.5)	0.0094 *
AK II	84 (37.8)	38 (34.2)	46 (41.4)	0.2693
AK III	49 (22.1)	19 (17.1)	30 (27)	0.0757
AK Basal Growth Grading				<0.0001 *
PRO 0	10 (4.5)	1 (0.9)	9 (8.1)	0.0098 *
PRO I	32 (14.4)	5 (4.5)	27 (24.3)	<0.0001 *
PRO II	54 (24.3)	16 (14.4)	38 (34.2)	0.0006 *
PRO III	126 (56.8)	89 (80.2)	37 (33.3)	<0.0001 *
Acantholysis	102 (45.9)	66 (59.5)	36 (32.4)	<0.0001 *
Elastosis				0.1107
Unknown	8 (3.6)	7 (6.3)	1 (0.9)	0.0311 *
None	17 (7.7)	17 (15.3)	0 (0)	0.0004 *
Mild	28 (12.6)	12 (10.8)	16 (14.4)	0.8507
Moderate	42 (18.9)	21 (18.9)	21 (18.9)	1.0000
Severe	70 (31.5)	30 (27)	40 (36)	0.1495
Very severe	57 (25.7)	24 (21.6)	33 (29.7)	0.1677
Follicular Involvement	170 (76.6)	93 (83.8)	77 (69.4)	<0.0001 *
Hyperkeratosis				<0.0001 *
None	15 (6.8)	3 (2.7)	12 (10.8)	0.0163 *
Mild	60 (27)	20 (18)	40 (36)	0.0026
Moderate	84 (37.8)	47 (42.3)	37 (33.3)	0.1674
Severe	39 (17.6)	26 (23.4)	13 (11.7)	0.0222 *
Very severe	24 (10.8)	15 (13.5)	9 (8.1)	0.1957
Infiltrate				0.4373
None	8 (3.6)	2 (1.8)	6 (5.4)	0.1507
Mild	73 (32.9)	44 (39.6)	29 (26.1)	0.0325 *
Moderate	98 (44.1)	44 (39.6)	54 (48.6)	0.1775
Severe	36 (16.2)	14 (12.6)	22 (19.8)	0.1461
Very severe	7 (3.2)	7 (6.3)	0 (0)	0.0073 *

* Significant differences (*p* < 0.05) between sOTRs and the ICG.

## Data Availability

The data presented in this study are available on request from the corresponding author.
